# Validated LC Method for the Estimation of Voriconazole in Bulk and Formulation

**DOI:** 10.4103/0250-474X.59558

**Published:** 2009

**Authors:** C. N. Patel, J. B. Dave, J. V. Patel, B. Panigrahi

**Affiliations:** Department of Pharmaceutical Chemistry, Shri Sarvajanik Pharmacy College, Near Arvind Baug, Mehsana-384 001, India

**Keywords:** Reverse phase liquid chromatography, voriconazole, specificity, validation

## Abstract

Reversed phase high performance liquid chromatographic method was developed and validated for the estimation of voriconazole in bulk and formulation using prominence diode array detector. Selected mobile phase was a combination of water:acetonitrile (35:65 % v/v) and wavelength selected was 256 nm. Retention time of voriconazole was 3.95 min. Linearity of the method was found to be 0.1 to 2 μg/ml, with the regression coefficient of 0.999. This method was validated according to ICH guidelines. Quantification was done by calculating area of the peak and the detection limit and quantitation limit ware 0.026 μg/ml and 0.1 μg/ml, respectively. There was no significant difference in the intra day and inter day analysis of voriconazole determined for three different concentrations using this method. Present method can be applied for the determination of voriconazole in quality control of formulation without interference of the excipients.

Voriconazole is designated chemically as (2*R*,3*S*)-2-(2,4-difluorophenyl)-3-(5-fluoro-4-pyrimidinyl)-1-(1H-1,2,4-triazol-1-yl)-2-butanol with an empirical formula of C_16_H_14_F_3_N_5_O, which is a novel broad spectrum antifungal agent and effective against Aspergillus, candidacies species[1–6]. Assay methods based on high-performance liquid chromatography with UV detection (HPLC–UV) have been developed to determine the concentration of voriconazole in human plasma[7]. Also LC method for quantification and separation of enantiomer of voriconazole is reported[8]. An existing HPLC assay method in human plasma was reported, however this method is based on size exclusion chromatography coupled on-line with a reverse phase HPLC system with column switching and is therefore complex[9,10]. Human pharmacokinetic data for voriconazole have been published[11]. A few methods were reported for the determination of voriconazole in human serum[12,13]. Reports regarding the determination of impurities[14] and separation of stereo isomers[15] also appear in literature. LC-MS has been successfully used to determine other antifungal agents in plasma.

The aim of the present work is to develop an accurate, selective, precise and robust RP-HPLC method for the determination of voriconazole and voriconazole in oral suspension powder. The proposed method was validated as per ICH guidelines[16,17] and its updated international convention[18].

A Shimadzu's HPLC (LC-2010 CHT), SDP-M20A prominence diode array detector and manual injector of 20-μl loop was used. Chromatograms were analyzed using winchrom software provided with the system. Separation was performed on a Kromasil 5 μ 100A CIB, 250×4.6 mm i.d., 5 micron 309811-6 phenomenex column. Mobile phase consisted of water:acetonitrile (35:65 v/v) was used. Flow rate was adjusted to 1.0 ml/min and wavelength was set to 256 nm.

Pure voriconazole was obtained as gift sample from Cipla Pharmaceutical Limited (Mumbai, India), acetonitrile and methanol (HPLC grade) were purchased from S. D. Fine Chemicals Ltd., Mumbai, India. Triple distilled water, nylon 0.45 μm-47 mm membrane filter from Gelman laboratory, Mumbai, India and formulation was procured from local pharmacy.

Voriconazole standard stock solution (10 μg/ml) was prepared by transferring accurately weighed 10 mg of standard voriconazole to 100 ml volumetric flask and dissolved in methanol. The volume was adjusted up to the mark with methanol. From this solution 5 ml was accurately transferred into a 50 ml volumetric flask and volume was made up to the mark with methanol.

Voriconazole suspension powder was accurately weighed. A quantity of powder equivalent to 10 mg of voriconazole was transferred in to a 100 ml of volumetric flask and mixed with methanol (50 ml) and sonicated for 20 min. The solution was filtered through Whatman filter paper No. 41 and the residue was washed thoroughly with methanol. The filtrate and washings were combined in a 100 ml volumetric flask and diluted to the mark with methanol (100 μg/ml). The solution (1.0 ml) was further diluted to the mark in 10 ml volumetric flask with mobile phase (10 μg/ml).

A calibration curve was plotted over a concentration range of 0.1-2 μg/ml for voriconazole. Accurately measured standard stock solution of voriconazole (0.1, 0.3, 0.5, 0.7, 0.9, 1.1, 1.5, 1.7 and 2.0 ml) was transferred to a series of 10 ml corning volumetric flasks and the volume in each flask was adjusted to 10 ml with mobile phase. The resulting solution was injected and the peak area obtained at retention time 3.95 min and flow rate 1.0 ml/min was measured at 256 nm. Calibration curve was constructed for voriconazole by plotting peak area versus concentration at 256 nm. Each reading was average of five determinations.

Aliquots of prepared sample (10 μg/ml), equivalent to 0.1-2 μg/ml voriconazole, were transferred to series of 10 ml volumetric flasks and procedure was completed as described under construction of calibration curve. The concentrations of voriconazole were determined from the regression equation, and the mean recoveries were calculated.

To optimize the HPLC parameters, several mobile phase compositions were tried. Satisfactory peak symmetry was obtained with a mobile phase consisting of water:acetonitrile (35:65 v/v). The quantitation was achieved with prominence diode array detector at 256 nm based on peak area. The retention time was found to be 3.95±0.12 min. The optimized chromatographic conditions are mentioned in Table 1. A representative chromatogram is shown in fig. 1. The linearity range obtained for voriconazole was 0.1-2 μg/ml. System suitability tests for HPLC were performed on freshly prepared standard stock solution of voriconazole.

**Fig. 1:**
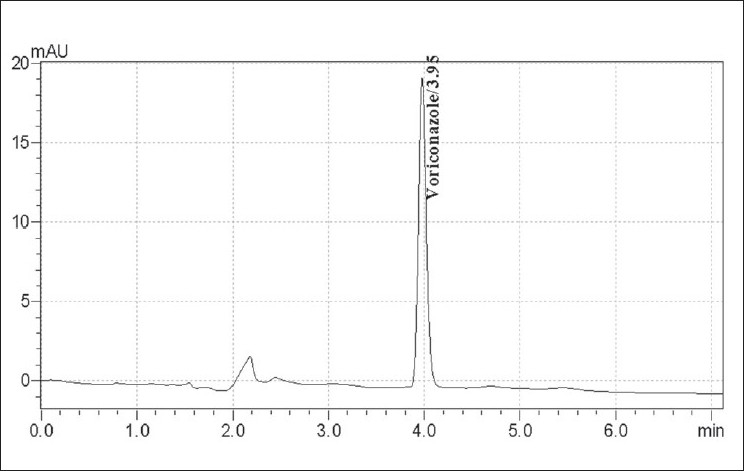
HPLC chromatogram of voriconazole Chromatogram of voriconazole with corresponding retention time at 256 nm.

**TABLE 1:** OPTIMIZED CHROMATOGRAPHIC CONDITIONS

Parameter	Description
Column	Kromasil 5 μ 100A CIB, 250×4.6 mm id, 5 micron 309811-6 phenomenex column
Mobile phase	Water:Acetonitrile::35:65% v/v
Detection	256 nm
Injection volume	20 μl
Flow rate	1.0 ml/min
Run time	8 min

Method precision was determined by preparing the standard solution of voriconazole (0.9 μg/ml) and analyzed 6 times as per the proposed method. Percentage relative standard deviation (% RSD) or coefficient of variation (CV) was not more than 2%. The intra-day precision (CV) was determined for standard solution of voriconazole (0.1-2 μg/ml) for 5 times on the day. The inter-day precision (CV) was determined for standard solution of voriconazole (0.1-2 μg/ml) for 5 days. The proposed method provides acceptable inter-day and intra-day variation for voriconazole at different concentration levels: 0.5, 0.9 and 1.5 μg/ml.

The proposed method for extraction and subsequent determination of voriconazole from pharmaceutical formulation after spiking with additional drugs afforded recovery values of 99.06–101.73 %. The developed method was used to quantify voriconazole in its formulation. All determinations were conducted five times. The system suitability and validation parameters are mentioned in Table 2.

**TABLE 2:** VALIDATION AND SYSTEM SUITABILITY PARAMETERS

Parameters	Results
Retention time	3.95 min
Tailing factor	0.96
Resolution factor	6.42
Theoretical plates	5643
Linearity range (μg/ml)	0.1-2
Correlation co efficient (r2)	0.999
Precision (% CV)	
Intra day (n=5)	0.37-0.63
Inter day (n=5)	0.69-01.33
Accuracy (% Recovery) (n=5)	99.08-101.73
Limit of detection (μg/ml)	0.026
Limit of quantification (μg/ml)	0.1
Specificity	Specific

The proposed validated method was successfully applied to determine voriconazole in bulk powder and in formulation. The estimated result of voriconazole in bulk and formulation are given in Table 3. The percent recoveries obtained was 99.06-101.73, indicates none interference from the common excipients in the formulation. It can be conveniently adopted for routine quality control testing of voriconazole in bulk and in is pharmaceutical dosage forms.

**TABLE 3:** ESTIMATION OF VORICONAZOLE IN FORMULATION

Formulations	Labeled/taken amount (mg)	Amount found (mg)	% Amount found ±SD (n=5)
Bulk powder	10	9.96	99.60±1.23
Formulation	60	60.73	101.21±1.26

The robustness and specificity of the LC method were established. Changes in the chromatographic system (column, mobile phase, pH, wavelength, and temperature) were used to evaluate the robustness. The retention time observed allowed a rapid determination of the drug, which is important for routine analysis. The use of mobile phase with out buffer which lengthen the column life is the main advantage for the proposed LC method. No interferences from excipients present in pharmaceutical formulation was observed at the detection wavelength. The chromatographic peak of analyte was not attributable to more than one component. At the same concentration, the peak areas of voriconazole standard and sample solutions were identical.

All these factors lead to the conclusion that the proposed method is accurate, precise, simple, sensitive, selective, robust and rapid and can be applied successfully for the estimation of voriconazole in bulk and in pharmaceutical formulations.
